# Extract from Dr. Benjamin W. Richardson’s Oration on the Vocation of the Medical Scholar, Delivered before the Medical Society of London

**Published:** 1856-07

**Authors:** 


*Extract from Dr. Benjamin W. Richardson's Oration on the Vocation of the Medical Scholar, delivered before the Medical Society of London.*

We are sometimes accused by the world of being prejudiced, and of being bound to certain dogmas, set phrases, systems, and ceremonials. We are not alone in this position; for no body of men were ever yet banded together for the most sacred objects without having similar inuendoes thrown into their teeth. But we feel these reproaches the more frequently, because they are the hues and cries of quacks, as every one knows.

Let a selfish, shallow, ambition-crazed German palm on the world, with great noise, a defunct dogma, and promise to cure diseases by thrusting nothing under heaven in the pretence of something potent over earth, down the throats of deluded suffering men, and when the monstrous foolery of the system and the practice is repudiated by the stern and clear common sense of the medical body, the impostor at once makes market of the repudiation, and by heavy and groaning appeals to honesty, justice, and such like homely requests, which go to the heart rather than the intellect, he positively wriggles his way into a kind of silly position, and founds a system which, being of the lowest human, is, of course, temporary and trumpery.

Or let another man start on no system at all but sheer impudence, falsehood, and the invention of some absurd nostrum, and lo! he finds it a splendid advertisement to open the campaign, and to keep it open, by attacks on the doctors, who, for their parts, do not think him worthy of regard either for good or for evil.

These attacks the medical scholar must bear. They will never injure his position if he meets them in the right way, and the right way is that which shall lead him to be candid in regard to his own deficiencies, laborious in filling them up, and honest and earnest in protecting that which he knows and feels to be true.

In the performance of these duties is hidden, indeed, the morality of medicine; and thus moving onwards through life, the medical scholar, whether considered in his universal or individual capacity, must in the end conquer, and stand approved. The simple faith of his science is the sign of the simple faith of his whole being. Mysteries he has none to unfold to a favored few; secrets, not one to carry to the grave. The great living world is this man's priest, the illimitable space his confessional; and if the book of his life were closed but for a moment, he would wish it blotted out for ever.—*Glasgow Medical Journal.*

